# Infrarenal high intra-abdominal testis: fusion of T2-weighted and diffusion-weighted magnetic resonance images and pathological findings

**DOI:** 10.1186/s12894-017-0254-y

**Published:** 2017-08-24

**Authors:** Seiji Hoshi, Yuichi Sato, Junya Hata, Hidenori Akaihata, Soichiro Ogawa, Nobuhiro Haga, Yoshiyuki Kojima

**Affiliations:** Department of Urology, Fukushima Medical University School of Medicine, 1, Hikarigaoka, Fukushima, 960-1295 Japan

**Keywords:** Cryptorchidism, Intraabdominal testis, MRI, Stem cell, Case report

## Abstract

**Background:**

Several recent reports have demonstrated that the preoperative sensitivity and accuracy of identifying and locating non-palpable testes increases with the use of conventional MRI, in addition to diffusion-weighted imaging (DWI). Therefore, pre-operative prediction of the presence and location of testes using imaging techniques may guide management of intra-abdominal testis. Fowler-Stephens orchiopexy is effective for treating patients with intra-abdominal testis; however, long-term testicular function after this procedure has not been clarified. We present a case of a high intra-abdominal testis located below the kidney, and discuss the usefulness of fusion view with T2-weighted and DWI images to make a diagnosis of high intra-abdominal testis and the pathological findings to predict future fertility potential.

**Case presentation:**

A 10-month-old boy was referred to the urology department for the management of non-palpable testis. We employed not only conventional MRI, but also DWI, to improve the diagnostic accuracy of non-palpable testes by MRI examination. The high-intensity mass-like structure below the kidney on the T2-weighted image and the markedly high signal intensity mass on the DWI image completely matched, which suggested that the mass below the kidney was the right testis. The patient underwent diagnostic and therapeutic laparoscopy. A testis was found under the ascending colon, 1 cm below the right kidney. We performed 2-stage Fowler-Stephens orchiopexy. The testis could be delivered to the scrotum without any tension. We examined expression patterns of the stem cell marker, undifferentiated embryonic cell transcription factor 1 (UTF1) in the testicular biopsy sample, and demonstrated that the UTF1-positive Ad spermatogonia / negative Ad spermatogonia ratio was lower in this patient than in boys his age with descended and inguinal undescended testes, indicating that spermatogonial stem cell activity may decrease remarkably in this boy.

**Conclusions:**

Fusion view with T2-weighted and DWI images may be a useful diagnostic modality for high intra-abdominal testes. Fowler-Stephens orchiopexy may provide blood supply to the testis but that might not be enough to achieve spermatogenesis.

## Background

The advent of laparoscopy has dramatically changed the diagnosis and treatment of non-palpable testis in children. Laparoscopy is an accepted procedure to investigate the presence and location, and to perform subsequent treatment, of intra-abdominal testis. Several recent reports have demonstrated that the preoperative sensitivity and accuracy of identifying and locating non-palpable testes increases with the use of conventional MRI, in addition to diffusion-weighted imaging (DWI) [1, 2]. Therefore, pre-operative prediction of the presence and location of testes using imaging techniques may guide management of intra-abdominal testis. Fowler-Stephens orchiopexy is effective for treating patients with intra-abdominal testis; however, long-term testicular function after this procedure has not been clarified. We present a case of a high intra-abdominal testis located below the kidney, and discuss the usefulness of fusion view, with T2-weighted and DWI images, to make a diagnosis of high intra-abdominal testis and the pathological findings to predict future fertility potential.

## Case presentation

A 10-month-old boy was referred to the urology department for the management of non-palpable testis. There was no significant familial or past history. A clinical examination demonstrated a left, well-positioned testis, 16 × 11 × 11 mm in size, and an empty right scrotum. No abnormality of the external genitalia was found. Physical examination could not confirm the presence of the right testis.

**Fig. 1:**
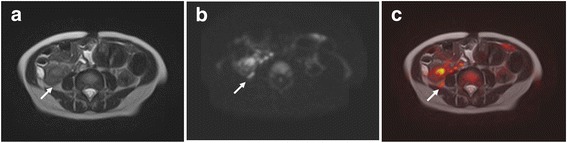
MRI findings. **a** T2-weighted image. **b** DWI. (**c**) T2-weighted image and DWI fusion image using medical image viewer software (EV Insite R, PSP Co., Tokyo, Japan). *Arrows*: intra-abdominal testis located below right kidney

Ultrasonography could not identify the right testis in the abdomen or inguinal region. We employed not only conventional MRI, but also DWI, to improve the diagnostic accuracy of non-palpable testes by MRI examination. The T2-weighted image showed a high-intensity mass-like structure, which was difficult to distinguish from the surrounding fat tissue, below the right kidney. Furthermore, DWI showed a mass, at that position, of markedly high signal intensity. The T2-weighted image was fused with the DWI image, using medical image viewer software (EV Insite R, PSP Co., Tokyo, Japan), to identify the anatomical location. The high-intensity mass-like structure on the T2-weighted image and the markedly high signal intensity mass on the DWI image completely matched (Fig. 1), which suggested that the mass below the kidney was the right testis.

**Fig. 2:**
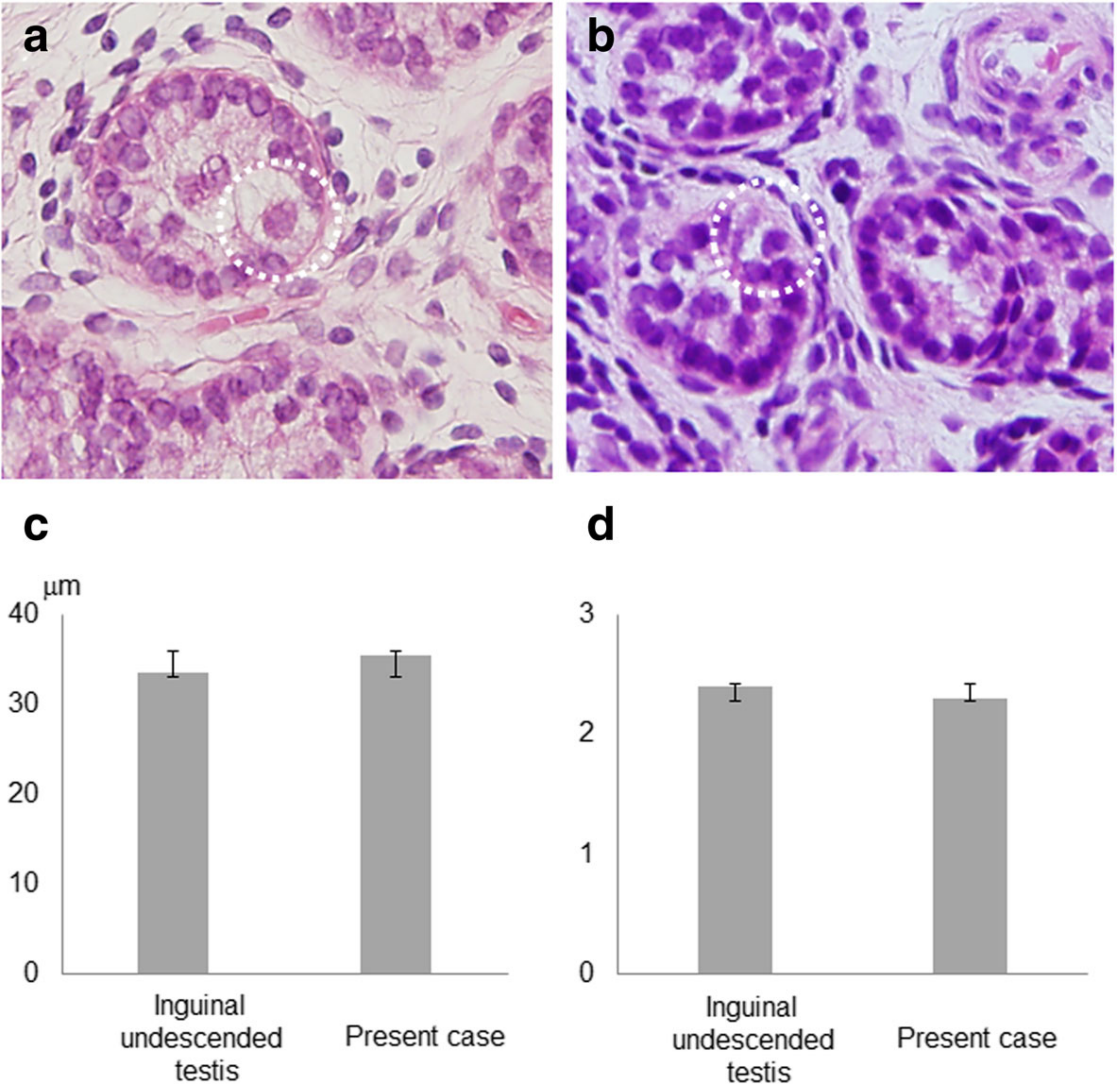
Histopathological findings of testicular tissue (hematoxylin-eosin staining; × 400) in inguinal undescended testis (**a**) and the present case (**b**). *Dotted circle*: spermatogonia. Median seminiferous tubule diameter (**c**) and median number of spermatogonia per tubular cross section (**d**) in inguinal undescended testis cases and the present case. *Error bars*: standard deviation

**Fig. 3:**
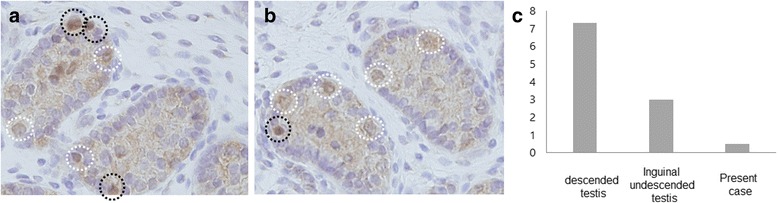
Comparison of UTF1 expression patterns in the testis between inguinal undescended testis (**a**) and the present case (**b**). *White dotted circle*: UTF1-negative Ad spermatogonia. *Black dotted circle*: UTF1-positive Ad spermatogonia. **c** The ratio of UTF1-negative Ad spermatogonia to UTF1-positive Ad spermatogonia in the testis among descended testis, inguinal undescended testis, and the present case

The patient underwent diagnostic and therapeutic laparoscopy. The 5-mm 0-degree camera, introduced through the umbilicus, showed an opened right inguinal ring, with vas deferens. Two more trocars were introduced and a testis was found under the ascending colon, 1 cm below the right kidney. We decided to perform 2-stage Fowler-Stephens orchiopexy. The spermatic vessels were ligated with nonabsorbable 2–0 sutures with the expectation that this would allow collateral blood supply to develop more fully. Ten months after the first surgery, the second stage of the 2-stage laparoscopic Fowler-Stephens procedure was performed. Because the vessel derived from the inferior epigastric artery had developed enough, as a feeding vessel, to provide blood supply to the right testis, retroperitoneal dissection was carried down from the level of the testis to the internal ring to create a wide peritoneal pedicle. The 15 × 10 × 6 mm testis could be delivered to the scrotum without any tension from the radially dilating system. After delivery of the testis, we performed testicular biopsy to compare pathological findings between the present patient and inguinal undescended testis, because we routinely do testicular biopsy during orchiopexy to predict future testicular function. For biopsy, the tunica albuginea was exposed and iris scissors were used to remove a small piece of the testicular parenchyma. Pathological findings in the biopsies taken at stage 2 of the operation showed that median seminiferous tubule diameter and median number of spermatogonia per tubular cross section were comparable to those in inguinal undescended testes of boys his age (*n* = 5; Fig. 2). We also examined expression patterns of the stem cell marker, undifferentiated embryonic cell transcription factor 1 (UTF1) [3–5], and demonstrated that the UTF1-positive Ad spermatogonia / UTF1-negative Ad spermatogonia ratio was lower in this patient than in boys of his age with descended testes (*n* = 5) and in inguinal undescended testes (the same biopsy samples used in the pathological study) (Fig. 3). This was part of a study examining the histopathological findings and stem cell activity of undescended testes, which was approved by the ethics committee of Fukushima Medical University School of Medicine. Informed consent was obtained from the patients before the study, after explaining its purpose and methods.

Ten months after the second surgery, the right testis, 13 × 8 × 7 mm in size, was located in the scrotum, and good vascularization was detected on echo color Doppler ultrasound.

## Discussion

Diagnostic modalities, such as ultrasound and MRI, are nonspecific in the intra-abdominal testis, and the accuracy of these examinations was not satisfactory. The only effective diagnostic procedure is considered to be laparoscopy. However, there is a possibility of being unable to locate a testis on diagnostic laparoscopy [6]. An accurate preoperative diagnosis is usually useful to determine the surgical approach for non-palpable testes management, particularly for high intra-abdominal cases. Kanrarci reported that identifying and locating non-palpable testes was improved by using the combination of DWI and conventional MRI, with about 90% sensitivity and accuracy [1]. Kato et al. also reported that preoperative combined assessment using T1- and T2-weighted imaging, fat-suppressed T2-weighted imaging, and DWI enabled the identification of intra-abdominal or intra-canalicular testes preoperatively and facilitated an accurate diagnosis of non-palpable testes [2]. They demonstrated that combined MRI assessments had an accuracy of 92.3% in the diagnosis of intra-abdominal testes; however, high intra-abdominal testes located over the pelvis, as in the present case, were not included in their reports [2]. In the present case, we could predict the presence and location of the intra-abdominal testis, below the kidney, preoperatively by fusion view with T2-weighted and DWI images. At our center, we usually perform MRI in patients who have a non-palpable testis without contralateral testicular hypertrophy and if ultrasonography fails to demonstrate a testis, because MRI improves the accuracy of diagnosing non-palpable testis and can be useful for identifying the presence and location of a testis. However, as described in the AUA guideline, although MRI is being used more widely due to greater sensitivity and specificity, it has the problems of higher cost, low availability, and need for anesthesia [7]. In addition, no imaging method can confirm absence of the testis with 100% accuracy [7]. Therefore, the AUA guideline recommends surgical exploration, such as diagnostic laparoscopy (or open exploration), rather than MRI [7]. Despite this recommendation, it would have been quite difficult to find the right testis at laparoscopy in our patient without the information obtained by MRI. Although it is hard to be dogmatic about the situations in which MRI is beneficial, examination of fusion views with T2-weighted and DWI images may be useful for preoperative localization of high intra-abdominal testes or for postoperative assessment in the rare cases where the testis cannot be identified by laparoscopy.

Previous reports demonstrated that the success rate of Fowler-Stephens orchiopexy, which was assessed by whether the testis was returned to the scrotum or the testicular size, was 69–95% [8, 9]. A recent report suggested that the testicular blood supply after Fowler-Stephens orchiopexy was preserved in most cases [10]; however, testicular function after Fowler-Stephens orchiopexy has not been clarified. Rosito et al. reported that ligation of the spermatic vessels during the first stage of orchiopexy for intra-abdominal testis was associated with a significant reduction of spermatogonia, although no significant changes were observed in the volumetric characteristics of the testes [11]. Kamisawa et al. also reported, in their animal study, that Fowler-Stephens orchiopexy may not significantly contribute to the improvement of spermatogenesis [12]. In our case, we examined the histological findings and expression pattern of the stem cell marker, UTF1, in the testicular biopsy specimen, during the second stage of a 2-stage Fowler-Stephens orchiopexy. A previous animal study demonstrated that the differentiation from gonocytes into early A spermatogonia and the stem cell activity of early A spermatogonia were disturbed during the early stage of spermatogenesis, suggesting that the loss of spermatogonial stem cell activity results in disturbances in spermatogenesis and may make fertility difficult in cryptorchidism [13]. In our patient, although good vascularization was detected on echo color Doppler ultrasound, and median seminiferous tubule diameter and median number of spermatogonia per tubular cross section were comparable to those in inguinal undescended testis cases, the UTF1-positive Ad spermatogonia (actual stem cells) / UTF1-negative Ad spermatogonia (potential stem cells) ratio was lower than in descended testes and inguinal undescended testes. This implies that spermatogonial stem cell activity was markedly reduced, and that development of collateral vessels after the first stage of 2-stage Fowler-Stephens orchiopexy may provide enough blood to maintain testicular viability but might not be sufficient to achieve spermatogenesis, although it is unclear if the testicular findings were attributable to the intra-abdominal location of the testis or were caused by ligation of the primary testicular vessels and the 10-month collateralization period.

## Conclusions

We presented a case of a high intra-abdominal testis located below the kidney. Fusion view with T2-weighted and DWI images may be a useful diagnostic modality for high intra-abdominal testes. Fowler-Stephens orchiopexy may provide blood supply to the testis but that might not be enough to achieve spermatogenesis; therefore, further studies are needed.

## Abbreviations

DWI: Diffusion-weighted imaging
UTF1: Undifferentiated embryonic cell transcription factor 1
